# Structure of a Single-Chain Fv Bound to the 17 N-Terminal Residues of Huntingtin Provides Insights into Pathogenic Amyloid Formation and Suppression

**DOI:** 10.1016/j.jmb.2015.03.021

**Published:** 2015-06-19

**Authors:** Erwin De Genst, Dimitri Y. Chirgadze, Fabrice A.C. Klein, David C. Butler, Dijana Matak-Vinković, Yvon Trottier, James S. Huston, Anne Messer, Christopher M. Dobson

**Affiliations:** 1 Department of Chemistry, University of Cambridge, Lensfield Road, Cambridge CB2 1EW, UK; 2 Department of Biochemistry, University of Cambridge, Tennis Court Road, Cambridge CB2 1GA, UK; 3 Translational Medicine and Neurogenetics Programme, Institute of Genetics and Molecular and Cellular Biology, 67404 Illkirch Cédex, France; 4 Neural Stem Cell Institute, Regenerative Research Foundation, Rensselaer, NY 12144, USA; 5 Department of Biomedical Sciences, University at Albany, Albany, NY 12208, USA; 6 James S. Huston, The Antibody Society, Newton, MA 02462, USA

**Keywords:** HD, Huntington's disease, HSQC, heteronuclear single quantum coherence, MBP, maltose binding protein, TEV, tobacco etch virus, CDR, complementarity-determining region, Huntington's disease, amyloid, single-chain Fv antibody, aggregation inhibition, prefibrillar intermediates

## Abstract

Huntington's disease is triggered by misfolding of fragments of mutant forms of the huntingtin protein (mHTT) with aberrant polyglutamine expansions. The C4 single-chain Fv antibody (scFv) binds to the first 17 residues of huntingtin [HTT(1-17)] and generates substantial protection against multiple phenotypic pathologies *in situ* and *in vivo*. We show in this paper that C4 scFv inhibits amyloid formation by exon1 fragments of huntingtin *in vitro* and elucidate the structural basis for this inhibition and protection by determining the crystal structure of the complex of C4 scFv and HTT(1-17). The peptide binds with residues 3–11 forming an amphipathic helix that makes contact with the antibody fragment in such a way that the hydrophobic face of this helix is shielded from the solvent. Residues 12–17 of the peptide are in an extended conformation and interact with the same region of another C4 scFv:HTT(1-17) complex in the asymmetric unit, resulting in a β-sheet interface within a dimeric C4 scFv:HTT(1-17) complex. The nature of this scFv–peptide complex was further explored in solution by high-resolution NMR and physicochemical analysis of species in solution. The results provide insights into the manner in which C4 scFv inhibits the aggregation of HTT, and hence into its therapeutic potential, and suggests a structural basis for the initial interactions that underlie the formation of disease-associated amyloid fibrils by HTT.

## Highlights

• C4 scFv specific for HTT(1-17) inhibits mHTT aggregation *in vitro*.
• HTT(1-17) binds to C4 scFv in a partly helical, partly extended conformation.
• C4 scFv increases the solubility of HTT(1-17) and sterically hinders mHTT aggregation.
• C4 scFv:HTT(1-17) crystallizes in a dimeric assembly forming an extended β-sheet network.

## Graphical Abstract

**Figure:**
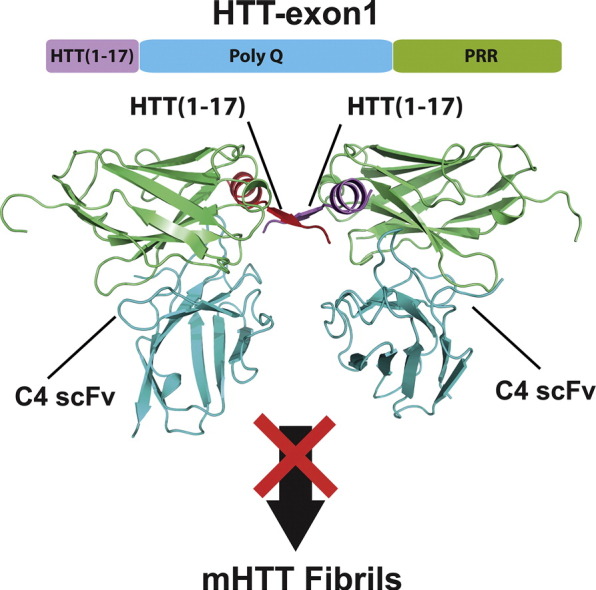

## Introduction

Huntington's disease (HD) is the most prevalent of a set of human neurodegenerative disorders linked to expansion of polyglutamine (polyQ) tracts in specific proteins [1,2]. The full-length protein associated with HD, huntingtin (HTT), is found predominantly in the cytosol. In the disease state, however, N-terminal proteolytic fragments of mutant forms of HTT (mHTT), which are characterized by aberrant expansions of the wild-type polyQ tract of > 36 residues, are found to accumulate as insoluble inclusions in cellular nuclei [3–6]. The proteolytic fragment of mHTT that is found to be most pathogenic is the exon1 fragment of mHTT, and overexpression of this fragment has been shown to be sufficient to produce rapid HD pathology in cells and organotypic tissue, as well as in yeast and animal models, including *Drosophila* and mouse models [7–10].

The sequence of the HTT-exon1 fragment can be divided into three regions: a 17-residue N-terminal region [HTT(1-17)], immediately followed by the polyQ tract of variable length and a proline-rich region at the C-terminal end of the peptide [11]. The HTT(1-17) region is highly conserved, has a high propensity to adopt an amphipathic α-helical structure and has been shown to be involved in membrane binding, sub-cellular localization, aggregation and toxicity [12–20]. The C- and N-terminal polyQ flanking sequences have opposite effects on the aggregation kinetics of mHTT-exon1 fragments when studied *in vitro*. Whereas the proline-rich sequence at the C-terminal end of the protein has a strong inhibitory effect on the aggregation of mHTT-exon1 fragments, the HTT(1-17) region enhances the aggregation kinetics of these fragments and of designed synthetic fusions of HTT(1-17) and other polyQ peptides by several orders of magnitude [21,22]. It has been suggested that the HTT(1-17) and the proline-rich regions interact with each other in the full-length protein and also that expansion of the polyQ chain disrupts this interaction, leading to a higher propensity for mHTT to aggregate [14].

A naïve human single-chain Fv antibody (scFv) phage-display library screened against HTT(1-17) was used to select the C4 scFv for use as an intrabody (a scFv antibody fragment that is engineered to be expressed within diseased cells). The C4 scFv intrabody can very effectively counteract the length-dependent polyglutamine expansion that induces pathologic HTT aggregation in cell culture, *Drosophila*, and mouse models of HD [23–25]. In this manuscript, we describe the effects of C4 scFv on the *in vitro* aggregation properties of mHTT protein fragments and report the crystal structure of the antibody fragment in complex with the 17-residue peptide at 2.5 Å resolution, as well as the characteristics of the binding of these two species in solution using NMR spectroscopy.

## Results

### Inhibition of the *in vitro* aggregation of mHTT-exon1 huntingtin fragments by the intrabody C4 scFv

**Fig. 1:**
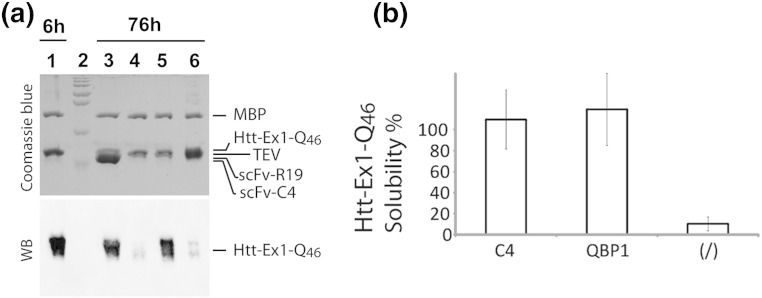
C4 scFv specifically inhibits the aggregation of the exon1 of mHTT *in vitro*. (a) Top panel: Coomassie-stained gel of samples of MBP-HTT-Ex1-Q46 after digestion with TEV for 6 h (lane 1) and a 6-h TEV digest of MBP-HTT-Ex1-Q46 subsequently incubated for 70 h (total time = 76 h) with C4 scFv (lane 3), with no added compound (lane 4), with QBP1 (lane 5) or with the negative control antibody fragment scFvR19 (lane 6). The positions of the different proteins on the gel are indicated on the right-hand side and lane 2 shows protein molecular weight markers (from bottom to top: 28, 36, 55, 72, 95, 130 and 250 kDa). Bottom panel: Western blot analysis of the soluble fraction of HTT-Ex1-Q46 for each sample using 1C2 anti-polyQ as the primary antibody [61]. (b) Densitometric based quantification of the solubility of HTT-Ex1-Q46 in the presence of C4 scFv (C4) and QBP1 or in the absence of added compound (/), following the same incubation protocol as in (a). The solubility of HTT-Ex1-Q46 following 6 h of digestion is used as 100% solubility reference point. The standard deviations calculated from two independent experiments are shown as error bars.

The antibody fragment C4 scFv has been shown to inhibit strongly the formation of intracellular inclusions of mHTT-exon1 fragments of huntingtin in cellular and animal models of HD [23–25]. These experiments were, however, conducted in complex cellular environments, and so we investigated the *in vitro* ability of the isolated C4 scFv protein to inhibit the aggregation of mHTT-exon1 protein fragments. Here, we used purified HTT-exon1 peptides that contain 46 glutamine residues in their polyQ tract (HTT-Ex1-Q46), which were expressed as recombinant and soluble maltose binding protein (MBP) fusion proteins in *Escherichia coli* [26]. Proteolytic removal of this MBP solubilization tag using tobacco etch virus (TEV) protease initiated the aggregation of the HTT-Ex1-Q46 peptide, which was essentially complete after ~ 76 h (Fig. 1).

When C4 scFv was added after the proteolytic cleavage of MBP, effectively all of the HTT-Ex1-Q46 peptide remained soluble for incubation times of at least 76 h, an effect similar to that of the well-known peptide inhibitor of polyQ aggregation, polyglutamine binding peptide 1, QBP1 [27], which we included in our experimental strategy as a positive control (Fig. 1). To evaluate the specificity of the inhibition of mHTT by C4 scFv, we incorporated a gankyrin-specific scFv, scFvR19 [28], into the experimental design. As expected, this antibody fragment had no detectable effect on the extent of aggregation by HTT-Ex1-Q46 when it was added at the beginning of the aggregation reaction (Fig. 1).

### Crystal structure of the C4 scFv:HTT(1-17) complex

We investigated the nature of the interaction of the C4 scFv intrabody with the HTT(1-17) peptide at atomic resolution by co-crystallizing the two species. The complex was found to crystallize under a range of conditions involving mixtures of polyethylene glycol 4000, ammonium sulfate and acetate buffer, at pH values close to 4.6. Several crystals were tested for their diffraction properties and we studied in detail the one showing the highest-quality data, for which the low-temperature X-ray diffraction data extended to 2.5 Å. Crystallographic analysis of the diffraction data further revealed that the crystal belonged to space group *C*2, with unit cell dimensions *a* = 151.3 Å, *b* = 35.9 Å, *c* = 110.95 Å, α = 90.0°, β = 120.72° and γ = 90.0° (Table 1). We solved the structure by using the maximum likelihood molecular replacement technique and a combination of automated and manual building and refinement steps. The structure was refined to give *R* and *R*_free_ factors equal to 18.2% and 22.6%, respectively (Table 1).

**Fig. 2:**
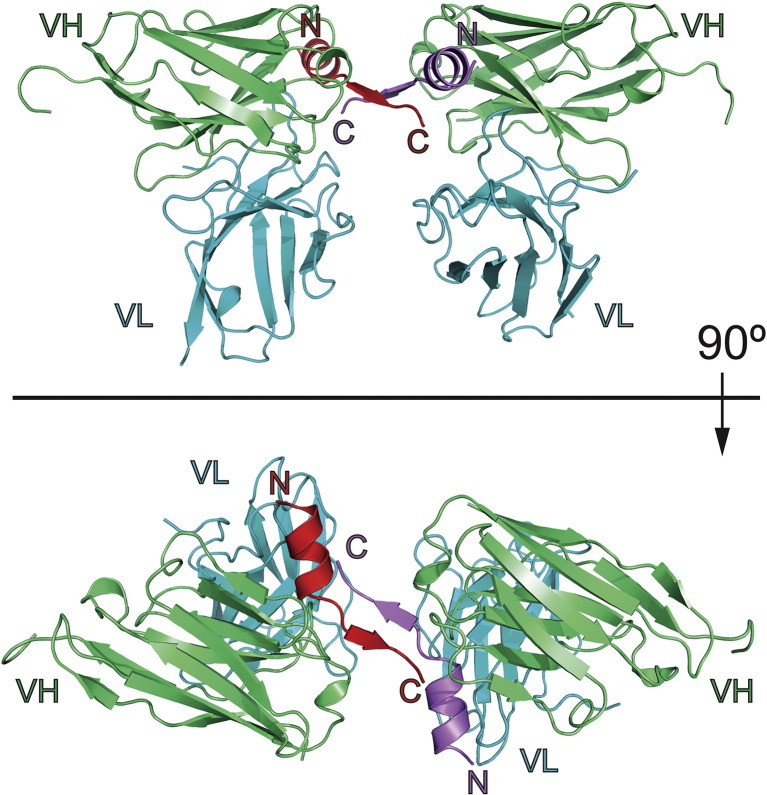
Crystal structure of the C4 scFv:HTT(1-17) complex. Ribbon diagram of the two C4 scFv:HTT(1-17) complexes in the asymmetric unit. The VH and VL domains of the C4 scFv are shown in green and blue, respectively (labeled “VH” and “VL” at the C-terminal side of the respective domain). The HTT(1-17) peptides are shown in red and purple and the N- and C-termini of the peptides are labeled with “N” and “C”. The top and the bottom view are related to each other by a 90° rotation along the *x*-axis.

The residues of the C4 scFv molecules were found to give rise to clearly defined regions of electron density, including those residues corresponding to the antigen binding loops L1–L3 and H1–H3. The loops L1 and L2 of the VL (λ) domain of C4 scFv were found to adopt conformations consistent with the canonical structure classes L_λ_1 and L_λ_2, respectively, and the conformation of the L3 loop could be classified as canonical structure class L_λ_3 1B [29]. The CDR-H1 and CDR-H2 loops [complementarity-determining region (CDR)] could be classified as canonical class 1 and canonical class 3, respectively [29]. We could also observe clear electron density for the atoms of the bound HTT(1-17) peptide, which enabled a structural model to be constructed (Fig. S1). We found that the asymmetric unit was in fact composed of two C4 scFv:HTT(1-17) complexes that were bound to each other through interactions between residues of the peptide ligand, resulting in a dimer of C4 scFv:HTT(1-17) complexes related to each other by a 2-fold non-crystallographic symmetry axis (Fig. 2).

**Fig. 3:**
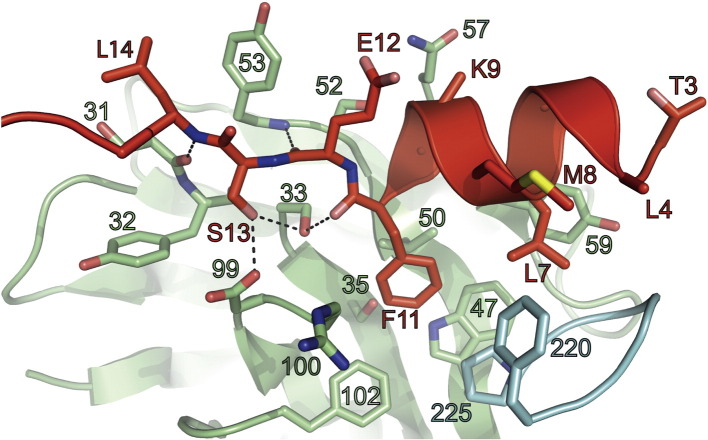
Detailed view of the binding interaction of a single C4 scFv molecule in complex with a single HTT(1-17) peptide. The VH and VL domains of the C4 scFv are shown in green and blue, respectively; the HTT(1-17) peptide is shown in red. The side chains of those residues of the HTT(1-17) peptide and of C4 scFv that have atoms that lie within 5 Å of each other are represented as sticks and are labeled. The hydrogen bonds made by residues 11, 12 and 13 of HTT(1-17), with residues of C4 scFv are shown as dotted lines.

**Fig. 4:**
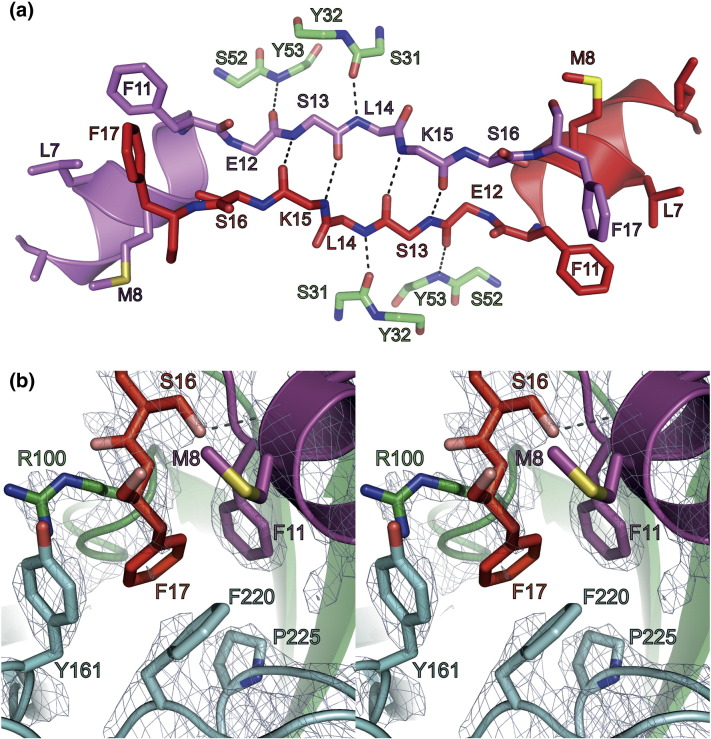
Detailed view of the interaction between the two peptides in the dimeric C4 scFv:HTT(1-17) complex. (a) General view and (b) stereo representation of the detailed view of the interaction of the side chain of F17 of HTT(1-17) with residues of the other HTT(1-17) peptide:C4 scFv complex in the asymmetric unit. The C atoms of the peptides bound to the different C4 scFv molecules are colored purple and red, respectively, and O and N atoms are colored red (pink) and blue, respectively. Residues originating from the antibody fragments are colorcoded as follows: C, cyan (VL) or green (VH); O, red; N, blue. Interacting residues are labeled and hydrogen bonds are indicated using gray dotted lines. 2*F*_o_ − *F*_c_ electron density maps (at 1.2σ) of the residues in this region are represented as a light-blue mesh.

Detailed analysis of a single C4 scFv:HTT(1-17) complex shows that the residues 3–11 of the bound HTT(1-17) peptide fold into a right-handed α-helix, while residues 12–17 of HTT(1-17) are in an extended conformation, of which residues 12–15 adopt a β-sheet structure (Figs. 3 and 4). The C4 scFv binding to the peptide involves the side chain of Phe11 of HTT(1-17) being located in a hydrophobic pocket formed by the side chains of Trp47^VH^, Val50^VH^, Pro225^VL^, Arg100^VH^ and Phe102^VH^ and the C^β^ atoms of residues Ser33^VH^ and Ser35^VH^ (Fig. 3). The side chain of Leu7^HTT^ makes contact with the aromatic ring of Tyr59^VH^ and Phe220^VL^ and interacts with the backbone atoms of several residues of the third antigen binding loop (CDR-L3) of the VL domain, namely, Asn222^VL^, Ser223^VL^ and Gly224^VL^. The O^γ^ atom of Ser33^VH^ is hydrogen bonded to the carbonyl oxygen of Phe11^HTT^, and the carbonyl oxygen of Glu12^HTT^ is hydrogen bonded to the NH backbone amide of Tyr53^VH^. Ser13^HTT^ forms hydrogen bonds with Asp99^VH^ and Ser33^VH^, and there is a main-chain hydrogen bond between the NH group of Leu14^HTT^ and the CO group of Ser31^VH^. Leu14^HTT^ also makes close contacts with the aromatic ring of Tyr53^VH^ (Fig. 3).

Of particular interest, however, is that the C4 scFv:HTT(1-17) complexes are bound together in pairs through interactions between residues 11 and 17 of both HTT(1-17) peptides. Indeed, the two peptide molecules associate to form an antiparallel β-sheet structure, involving an extensive network of hydrogen bonds between the main-chain atoms of residues 14–16 of each molecule (Fig. 4a), which is further stabilized through two C4 scFv-mediated hydrogen bonds between Ser31^VH^ and Leu14^HTT^ and between Tyr53^VH^ and Glu12^HTT^, resulting in an extended β-sheet (Fig. 4a). The amide and carbonyl groups of Ser13^HTT^ are hydrogen bonded to the carbonyl and amide groups of Lys15^HTT^, respectively, which in addition allows the O^γ^ of Ser16^HTT^ to make a hydrogen bond with the carbonyl group of Met8^HTT^ of the adjacent peptide molecule (Fig. 4b). The dimeric arrangement of both peptides is possibly also further stabilized by the burial of the side chain of Phe17^HTT^ in the hydrophobic cavity formed by the aromatic side chains of Phe11^HTT^, Phe220^VL^ and Tyr161^VL^ and by the aliphatic atoms of the side chain Arg100^VH^ (Fig. 4b). The side-chain atoms of both F17^HTT^ and F220^VL^ do not have well-defined positions in the electron density maps (Fig. 4b), suggesting that these residues are partially disordered and have a higher degree of conformational flexibility compared to the remainder of the structure.

To evaluate the significance and the possible biological relevance of the various interfaces found within the crystal structure, we performed PISA (Proteins, Interfaces, Structures and Assemblies) analysis (Table 2 and Table S1) [30]. This approach provides estimates for a number of parameters defining each interface from the details of the structure, including the free energy of formation, the gain in solvation energy, the total and buried areas of the interface, the number of hydrogen bonds and salt bridges across the interface and the hydrophobic complementarity [31]. Based on this analysis, potentially biologically relevant elements of quaternary structure can be distinguished from simple crystallographic contacts.

The free energies of solvation and the dissociation energies indicate that the interaction between the two C4 scFv:HTT(1-17) complexes in the asymmetric unit is likely to have a high stability in solution and suggests that the [C4 scFv:HTT(1-17)]_2_ dimer is the most significant assembly of the protein chains (Table 2). Table 2 further indicates that the interface between the peptide moieties in the C4 scFv:HTT(1-17) dimer, in particular, is expected to have the highest stability and the highest energetic cost for its dissociation of all the interfaces found in this assembly state.

### The nature of the C4 scFv:HTT(1-17) complex in solution

To probe the interaction of the C4 scFv with HTT(1-17) in solution, we performed gel-filtration chromatography, analytical centrifugation and mass spectrometry on samples containing either the intrabody or the peptide separately, or samples containing various amounts of both molecules (Fig. S2). The results of these experiments confirmed that both free C4 scFv and C4 scFv bound to the HTT(1-17) peptide are predominantly monomeric in solution (Fig. S2).

Using ^15^N-^1^H heteronuclear single quantum coherence (HSQC) NMR spectroscopy, we could probe in detail the binding of the HTT(1-17) peptide in solution by measuring the perturbations of the backbone amide resonances of the antibody fragment that resulted from the presence of HTT(1-17).

A series of standard three-dimensional triple-resonance NMR techniques enabled us to assign 201 and 208 of the 235 non-proline C4 scFv residues for the free and bound states of C4 scFv, respectively. We could not observe any peaks corresponding to the six histidine residue purification tag at the C-terminus of the C4 scFv and we could not assign 8 residues of the 12-residue Gly-Ser linker that connects the VH with the VL domain due to spectral overlap. Other “missing” resonances for both the free spectrum and the bound spectrum include G237, T203, I204, I177, Y178, G157, T152, Y101, Y95, V50, I51, V37, T28, G16 and Q5. In the free spectrum of the C4 scFv, the resonances corresponding to residues R100, D99, A97, C96, A61, Y60, Y59 and S52 could not be observed due to line broadening.

**Fig. 5:**
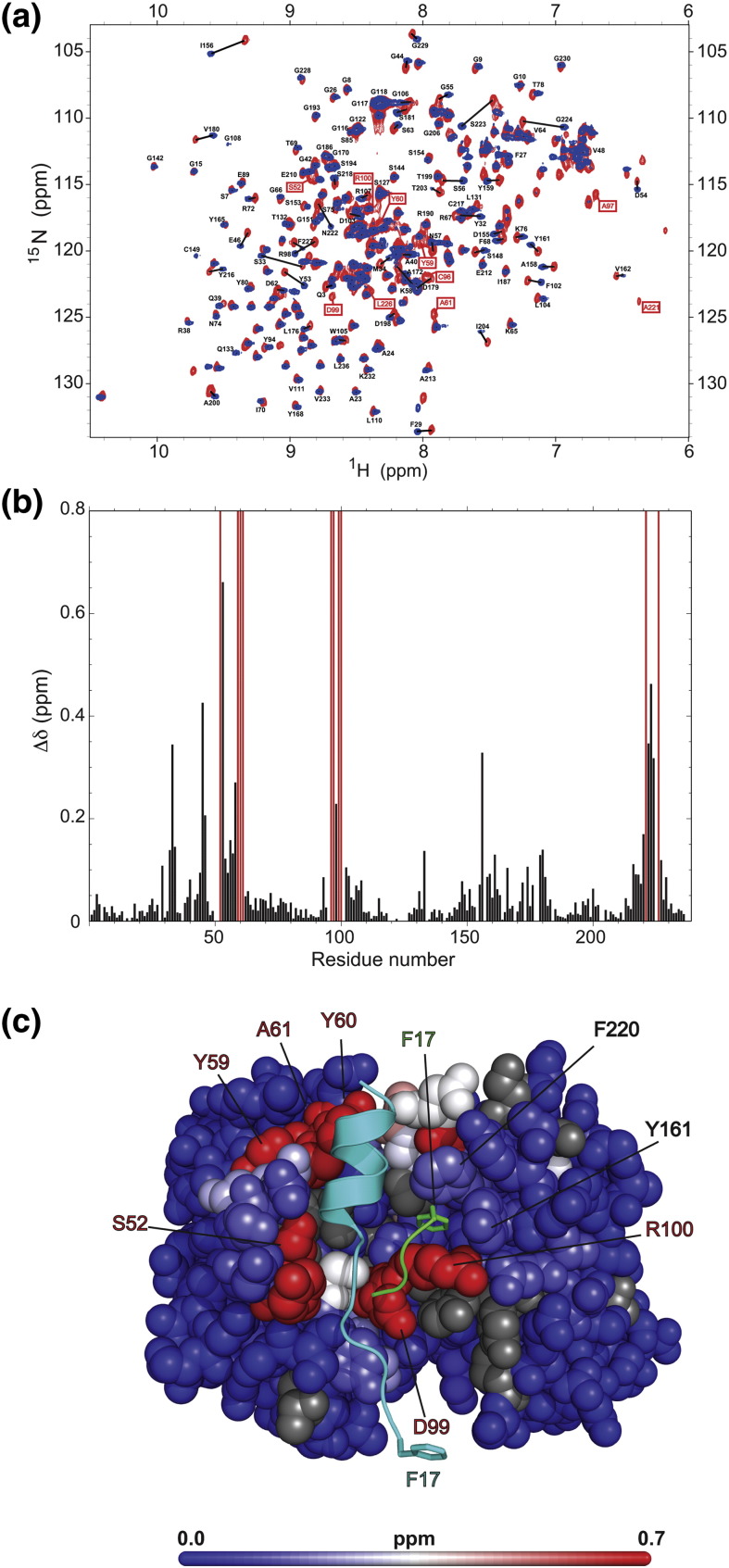
^15^N-^1^H Heteronuclear single quantum correlation spectroscopy measurements of uniformly labeled C4 scFv in its free form and bound to HTT(1-17), in solution. (a) Overlay of ^15^N-^1^H HSQC NMR spectra of uniformly ^15^N-labeled C4 scFv (red) and of uniformly ^15^N-labeled C4 scFv in the presence of 2 equivalents of unlabeled HTT(1-17) peptide (blue). Resonances of residues that show significant chemical shift differences between the free and the bound states are connected with a black line and resonances of residues that are not detectable in the free spectrum but become detectable in the bound spectrum are labeled in red boxes. (b) Bar graph representation of the chemical shift changes in the free and bound states for each of the assigned residues of C4 scFv (the change in chemical shift is defined as [0.04 × (δ^15^N_free_ − δ^15^N_bound_)^2^ + (δ^1^H_free_ − δ^1^H_bound_)^2^]^1/2^[66]). Resonances of the residues that could not be assigned in the free spectrum but that did become detectable in the bound state are drawn as red bars with an arbitrary *y* value of 0.8. (c) Values of the bar graph in (b) mapped on to the structure of C4 scFv in complex with the peptide HTT(1-17); the magnitudes of the shifts of C4 scFv residues are colorcoded going from dark blue (insignificant shift, ~ 0 ppm) to red (major shift, > 0.7 ppm) according to the colorcoding on the spectrum bar at the bottom of the panel. The residues indicated in red in (a) and (b) are also colored red on the structure and are labeled in red. The residues Y161 and F220, which show significant chemical shift perturbations and which are in contact with F17^HTT^ in the crystal structure, are also labeled. Unassigned residues in both spectra are colored gray, and the peptide is represented in ribbon format and colored cyan. The peptide residues 15^HTT^–17^HTT^ from the second C4 scFv:HTT(1-17) complex that make contact in the asymmetric are shown as a green ribbon. The side chain of Phe17^HTT^ in both peptides is also shown and labeled.

An overlay of the HSQC spectra measured for C4 scFv in its free state and in the presence of a saturating concentration of HTT(1-17) shows that many of the backbone amide resonances in the spectrum of free C4 scFv have shifted to new positions in the spectrum of the bound state (Fig. 5a), indicating that these residues are in close proximity to the site of peptide binding. In addition, the resonances of residues R100, D99, A97, C96, A61, Y60, Y59 and S52, for which the intensities of the signals in the free spectrum were below the noise level and that were therefore not assigned, could be detected and assigned in the bound spectrum. These residues are located in, respectively, the CDR-H2 and CDR-H3 regions of the C4 scFv and make contact with the HTT(1-17). The results suggest that these residues become more ordered in the bound state compared to the free state of the antibody fragment.

The residues whose resonances have the largest chemical shift perturbations coincide with the residues that are observed in the crystal structure to be involved in contacts with residues of the peptide (Fig. 5b and c). Small chemical shift perturbations are also observed for those residues, including Phe220^VL^ and Tyr161^VL^ of C4 scFv, that are in contact with Lys15^HTT^, Ser16^HTT^ and Phe17^HTT^ in the crystal structure, indicating that such contacts might also be formed in solution (Fig. 5b and c). There is no evidence, however, for line broadening associated with the formation of a higher-molecular-weight species corresponding to a dimeric arrangement of two C4 scFv:HTT(1-17) complexes. These observed shifts might therefore be explained by contacts between residues of the same peptide and the resulting contacts of these residues with the C4 scFv antibody fragment or by secondary perturbations of a stronger interaction located further away in the binding interface. As a preliminary evaluation of the contributions of residues Lys15^HTT^, Ser16^HTT^ and Phe17^HTT^, we performed isothermal calorimetry measurements using the wild-type peptide HTT(1-17) and the truncated peptides HTT(1-16), HTT(1-15) and HTT(1-14) (Fig. S3). Our observations show that the shorter peptides have slightly lower affinities, with the HTT(1-14) peptide showing a 10-fold decrease in binding affinity compared to HTT(1-17). Although this observation argues that Lys15^HTT^, Ser16^HTT^ and Phe17^HTT^ contribute to the binding of C4 scFv, the interaction appears to be weak and is probably highly dynamic.

## Discussion

### C4 scFv inhibition of mHTT-exon1 aggregation

In the present study, we have found that C4 scFv inhibits the aggregation of HTT-Ex1-Q72 peptides *in vitro* (Fig. 1), a result that is consistent with observations from *in situ* and *in vivo* studies [24,25]. The crystal structure of the intrabody C4 scFv in complex with the HTT(1-17) peptide determined in the present work provides a structural explanation for this inhibition as the binding of the C4 scFv to the peptide segment would sterically hinder the self-association process of the HTT(1-17) segment. Furthermore, C4 scFv increases the solubility of the peptide by interacting with the residues Leu4^HTT^, Leu8^HTT^ and Phe11^HTT^, which adopt helical conformations resulting in the formation of a hydrophobic surface. This surface is thus shielded from the solvent and protected from self-association with other HTT fragments, as well as from interactions with cellular membranes that have been suggested to be an important factor in the nucleation of toxic mHTT aggregates [18,32–35].

### The binding of C4 scFv to HTT(1-17) in solution

The intrabody C4 scFv was obtained from a human synthetic scFv library, and it was selected against a C-terminally biotinylated HTT(1-17) peptide [24]. The conformation of the HTT(1-17) peptide that is recognized by the C4 scFv is therefore expected to be one that is highly populated, or readily accessible, in solution as antibodies or antibody fragments do not generally bind to high-energy states [36]. Indeed, in recent studies including molecular dynamics simulations it was predicted that structures of HTT(1-17) in which residues 3–11 adopt α-helical conformations and residues 12–17 extended and disordered conformations are highly populated within the ensemble of solution structures [37]. The propensity of the HTT(1-17) peptide to form α-helical conformations has been confirmed by a range of biophysical studies, including CD, Fourier transform infrared (FTIR) spectroscopy and NMR spectroscopy, which show that the peptide is predominantly disordered in dilute solutions but that it acquires helical structure in concentrated solutions [38]. Similar increases in helical content can be induced by the presence of organic solvents, such as trifluoroethanol [34], or through interactions with other partners, such as detergents or membranes [18,34], or proteins, including molecular chaperones [7,39–41].

The major involvement of the α-helical region of HTT(1-17) in binding to a protein molecule is also revealed from the crystal structure of this peptide including the first residue of the polyQ domain of HTT, with a different intrabody, VL12.3 [42]. The binding site of this intrabody to HTT, however, involves residues 5^HTT^–18^HTT^ and so includes the first residue of the polyQ tract of HTT-exon1. In addition, all the residues of the peptide involved in binding to VL12.3 adopt an α-helical conformation (Fig. S4), most probably because a large region of exposed hydrophobic surface allows the burial in the complex of the hydrophobic side chains of Leu7^HTT^, Phe11^HTT^, Leu14^HTT^ and Phe17^HTT^, and may stabilize Gln18^HTT^ in a helical conformation.

### The origin and relevance of the dimeric arrangement of C4 scFv:HTT(1-17) complexes observed in the crystal structure

The remarkable dimeric C4 scFv:HTT(1-17) complex that is observed in the crystal structure (Fig. 2) could not be detected in solution by analytical centrifugation, gel filtration or mass spectrometric techniques (Fig. S2). The interactions involved in the formation of this complex are, however, extensive and are predicted to be highly relevant by PISA analysis (Table 2).

A question that emerges, therefore, is whether the intermolecular interaction found in the crystal is a newly formed interface during the crystallization process or if it is the result of domain swapping [43–45]. In the latter case, structurally and energetically equivalent “intra”-molecular interactions are exchanged with “inter”-molecular ones. This would imply that residues 15^HTT^–17^HTT^ fold back onto residues 11^HTT^–13^HTT^, in solution, generating favorable interactions within the same peptide molecule and interactions with the C4 scFv and possibly including an interaction of the side chain of Phe17^HTT^ in a hydrophobic pocket formed by the aromatic side chains of Phe220^VL^ and Tyr161^VL^ and the side chain of Arg100^VH^ (Fig. 4b). In our NMR analysis, small chemical shift perturbations for residues of C4 scFv that are in contact with residues 15^HTT^–17^HTT^ in the crystal structure that might be indicative of an interaction were observed (Fig. 5). Preliminary analysis of this interaction using isothermal calorimetry and truncated HTT(1-17) peptides shows, however, that this region contributes only moderately to the binding energy (Fig. S3). The elucidation of the exact roles of these residues in the binding interaction will, therefore, require further investigation.

Nevertheless, the formation of β-sheet structure within the C4 scFv:HTT(1-17) complexes, which primarily involves residues 11–17 of the HTT peptide, is a very interesting observation. It seems that, in this particular conformation of the peptide bound to the C4 scFv, residues 3^HTT^–11^HTT^ form an amphipathic helix and that residues 12^HTT^–17^HTT^ form an extended (β-sheet) structure, which seem to be stabilized by backbone hydrogen-bonding interactions with residues of the C4 scFv on either side of the antiparallel β-sheet formed by the residues 12^HTT^–17^HTT^ (Fig. 4a). It is therefore possible to speculate about the relevance of this conformation in the mechanism of enhanced mHTT fibril formation by HTT(1-17) *in vitro* and *in vivo* as the formation of this structural feature, if it were not blocked from further growth due to the presence of C4 scFv, might lead to a dramatically enhanced propensity of HTT to form β-sheet fibrillar structures. Interestingly, it has been shown from solid-state NMR measurements that this exact conformation of HTT(1-17), adopting a partly helical (residues 4–11) and a partly extended or β-sheet conformation (residues 12–17), is highly populated in mHTT fibrils [22]. The dimeric structure of C4 scFv:HTT(1-17) complex might therefore provide a clue as to the key initial events in HTT fibril formation.

### Significance for an antibody-bound therapeutic strategy targeting HD

Antibody technologies have proved to be capable of generating powerful therapeutic agents for a wide variety of diseases ranging from cancer to multiple sclerosis [46,47]. Increasing attention has recently been given to intrabodies [48,49], antibody fragments that are recombinantly engineered to be expressed in cells and that have exquisite specificity combined with high affinity, stability and solubility. Intrabodies have been shown to have significant potential for acting as inhibitors of events associated with protein misfolding and aggregation diseases [50–55]. For HD, several recombinant antibodies and intrabodies have been raised against translation products of HTT exon1, and it is now apparent that the ability of anti-HTT intrabodies to modulate aggregation and its associated neurotoxicity depends strongly on the epitope of HTT toward which they are directed [48].

The key involvement of the HTT(1-17) region in the pathogenic deposition of mHTT aggregates, as well as its inhibition by molecular chaperones that can bind HTT(1-17) [7,41,56], makes it a very attractive target for the development of intrabodies designed to suppress mHTT aggregation. At the present time, two intrabodies that bind to this region, VL12.3 [42] and C4 scFv [24], have been reported, and we have described the crystal structure of the latter when complexed with HTT(1-17) in the present manuscript. Both antibody species have been found to bind to the N-terminal residues of HTT and also to reduce mHTT-induced aggregation and toxicity very potently both *in vitro* and *in situ*, while not altering significantly the turnover of mHTT [24,25]. These intrabodies differ in the residues of HTT(1-17) with which they make contact and they have similar binding affinities, 44 nM (VL12.3) [57] *versus* 50 nM (C4 scFv) (Fig. S3). The epitope for VL12.3 is located further from the N-terminus of HTT(1-17) and appears to block cytoplasmic retention of mHTT-exon1 leading to much higher levels of an antigen–antibody complex in the nucleus [58,59] than are found for C4 scFv. The nucleus is known to be a major site of mHTT pathogenesis, and retention in the nucleus is likely to be at least partly responsible for the lack of long-term *in vivo* efficacy of this construct.

It has also been shown that binding of this scFv intrabody to full-length HTT is very weak, possibly due to a different conformation of the epitope when embedded in the full-length protein [25]. As a consequence of the latter effects, the presence of the intrabody in the cell is likely to have only minimal effects on the normal function of full-length HTT. To further enhance therapeutic efficacy of C4 scFv, we greatly increased degradation of toxic mHTT fragments by engineering the C4 scFv to include a proteasome degradation tag [23]. Thus, the combined properties of the C4 scFv, notably its cytoplasmic localization, its specificity for toxic HTT fragments and its amenability toward protein engineering, suggest that this intrabody could be a valuable lead in the quest for an effective therapeutic strategy for combating HD.

## Materials and Methods

### Cloning, expression and purification of C4 scFv

The gene for C4 scFv was synthesized (GenScript, New Jersey, USA) on the basis of the amino acid sequence of the protein (GenBank ID: ACA53373.1) and optimized for *E*. *coli* codon usage and cloned, expressed and purified as described hereafter. After the removal of the PstI restriction site at 628 bp, the gene for C4 scFv with the addition of a C-terminal tag containing six consecutive histidines to enhance purification was subcloned using PstI and EcoRI into the vector pHEN6 for expression in the periplasm of *E*. *coli* cells. Subsequently, CaCl_2_-competent WK6 *E*. *coli* cells were transformed with the recombinant DNA to obtain expression clones of the C4 scFv product. Isotopically naturally abundant, as well as ^15^N and ^13^C isotopically enriched, samples of C4 scFv were produced and purified according to previously reported protocols for periplasmic expression of single-domain antibodies [53].

### *In vitro* aggregation experiments

*In vitro* aggregation studies of the exon1 of the huntingtin protein carrying a stretch of 46 glutamine (HTT-Ex1-Q46) were performed using HTT-Ex1-Q46 fused to MBP (MBP-HTT-Ex1-Q46), purified as described earlier [26]. To induce amyloid formation by HTT-Ex1-Q46, we removed the MBP solubilization tag by incubation for 6 h with the TEV protease, which recognizes a specific cleavage site within the linker sequence between the MBP and HTT(1-17). At this 6-h timepoint, the MBP tag was fully removed, and the amount of soluble HTT-Ex1-Q46 was used as the 100% solubility reference. To assess the effect of different compounds on the aggregation of HTT-Ex1-Q46, we added these compounds after 6 h of initial TEV digestion, and the mixture was further incubated for 70 additional hours (total time, 76 h).

The detailed protocol was as follows:. 5 μl of MBP-HTT-Ex1-Q46 [7.6 μM, conserved at − 20 °C in 5 mM Tris, 25 mM NaCl, 25 mM KCl and 50% glycerol (pH 7.3)], 1 μl of TEV buffer [10 × stock: 500 mM Tris, 5 mM EDTA and 10 mM DTT (pH 8)], 1 μl of acTEV (stock: 10 U/μl; Invitrogen) and 1 μl of phosphate buffer (50 mM, pH 7) were mixed for 6 h at room temperature (~ 25 °C). After 6 h of digestion, 2 μl of either phosphate buffer, C4 scFv, QBP1 [60] or a non-relevant scFvR19 raised against gankyrin [28] was added and further incubated for different times (between 0 min for the point 6 h and 70 h for the point 76 h). Final concentrations upon incubation were as follows: MBP-HTT-Ex1-Q46 (3.8 μM), QBP1 (100 μM), C4 scFv (11 μM) and scFvR19 (11 μM). After incubation, samples were centrifuged (22,000*g*, 5 min, 4 °C) and 9.5 μl supernatant was mixed with 9.5 μl Laemmli loading dye and SDS-PAGE running buffer. We loaded 9 μl or 3 μl of each sample on several SDS-PAGE (16%) gels that, respectively, were either stained with Coomassie blue after electrophoresis or transferred onto nitrocellulose membranes for Western blotting with the primary 1C2 polyQ-specific antibody [61] and a secondary GAM-peroxidase antibody. Supersignal West Pico (Thermo Scientific, Illkirch, France) was added for revelation. The chemiluminescent signal was recorded on a Fusion (Fx7) image acquisition system, and the signal intensity was quantified using the included Bio1d software (Vilber Lourmat, Marne-La-Vallée, France). Note that QBP1, C4 scFv, scFvR19 and TEV are not detected by Western blotting using the 1C2 and GAM-peroxidase antibodies.

### Crystallization, X-ray data collection and structure solution

The C4 scFv antibody at 15 mg/ml was mixed with a 5-fold excess of a solubilized freeze-dried HTT(1-17) peptide in 20 mM Hepes buffer at pH 7.4 containing 150 mM NaCl. Crystals were grown by hanging-drop vapor diffusion in 0.2 M ammonium sulfate, 25% polyethylene glycol 4000 and 0.1 M sodium acetate buffer at pH 4.6.

X-ray diffraction data at 100 K of frozen C4 scFv:HTT(1-17) crystals were collected to a resolution of 3.1 Å on a Microstar microfocus rotating anode X-ray generator Icarus: X8 Proteum (Bruker AXS, Coventry, UK) equipped with a Platinum 135 CCD detector. The data were processed using Proteum 2 software (Bruker AXS) and the phase information associated with the structure factors was found by using the program Phaser [62]. The VL and VH domains of the crystal structure of the anti-SARS spike protein receptor domain scFv, 80R (2ghw) [63], after deletion of the coordinates for the residues of the hypervariable loop regions, were used as search models for the VL and the VH domains of the C4 scFv, respectively. Synchrotron X-ray diffraction data were recorded using the Proxima 1 beamline at the European Synchrotron Radiation Facility at SOLEIL in Paris, France. Data were collected to a resolution of 2.5 Å. Rebuilding of the hypervariable loops and the building of the peptide antigen structure was performed both manually and automatically using the program Auto-build in Phenix [64]. Phenix was also used to refine the structure. Data collection and refinement statistics are listed in Table 1.

### Structural analysis

The programs Contact, HBplus and Areaimol (Collaborative Computational Project Number 4) were used to analyze the structural and chemical features of the complex, namely, inter-atomic distances, hydrogen bonds and changes in solvent-accessible surface area upon complex formation. The Web server PISA† was used to score the biological significance of the various interfaces formed by the protein chains within the crystal [30]. Figures were prepared using the program PyMOL‡.

### NMR spectroscopy

Standard ^15^N-^1^H HSQC experiments were carried out at a ^1^H frequency of 700 MHz. Spectra of ^15^N-labeled C4 scFv were recorded free or in the presence of 1.2 molar equivalents of unlabeled HTT(1-17) peptide (Genemed Synthesis Inc., New York, USA). All experiments were recorded in 10 mM phosphate and 150 mM NaCl at pH 5.9 and at 310 K. For backbone assignments of C4 scFv in its unbound state, standard triple resonance experiments [HNCA, CBCA(CO)NH, HNCACB, HNCO and HN(Ca)CO spectra] were recorded at 310 K using a Bruker Avance 700-MHz spectrometer equipped with a cryogenic triple resonance probe (Bruker, Coventry, UK). The chemical shifts of individual spin systems (HN, N, Ca, Cb and CO) were collected manually and the backbone resonance assignments were achieved iteratively through a combination of computer-aided automated assignment procedure using the program Mars [65]. HNCA and CBCA(CO)NH spectra were recorded to determine the assignments of the resonances of the N, H, Ca and Cb atoms of the residues of C4 scFv in the presence of an equimolar amount of unlabeled HTT(1-17) peptide.

### Accession numbers

Coordinates have been deposited in the Protein Data Bank under ID code 4RAV.

## Figures and Tables

**Table 1:** Crystallography data and refinement statistics

Data collection C4 scFv:HTT(1-17)	
Space group	*C*2

*Cell dimensions*	
*a*, *b*, *c* (Å)	151.31, 35.93, 110.95
α, β, γ (°)	90.00, 120.72, 90.00
Resolution range (Å)^a^	44.24–2.50 (2.59–2.50)
*R*_merge_^a^	0.0305 (0.2542)
〈*I*/σ(*I*)〉^a^	14.45 (2.62)
Completeness (%)^a^	99.02 (96.97)
Redundancy^a^	4.6
Reflections measured^a^	35,795 (3484)
Unique reflections^a^	18,104 (1763)
Wilson *B*-factor (Å^2^)	44.87

*Refinement*	
Resolution range (Å)	44.24–2.50
Reflections (total)	18,092
Reflections (*R*_free_)	907
*R*_work_/*R*_free_ (%)	18.2/22.6
NCS groups	3
TLS groups	46
No. of non-hydrogen protein atoms	3554
No. of ligand atoms	20
No. of water molecules	32
Average *B*-factor (Å^2^)	53.60

*RMS deviations*	
Bonds (Å)	0.003
Angles (°)	0.72

*Ramachandran plot*	
Ramachandran favored (%)	96

a Numbers in brackets represent the highest-resolution shell.

**Table 2:** PISA analysis of the assemblies in the crystal structure of C4 scFv:HTT(1-17) that are expected to be stable in solution

Composition^a^	ASA^b^ (10^3^ Å^2^)	ΔASA^c^ (10^3^ Å^2^)	Δ*G*_int_^d^ (kcal/mol)	Δ*G*_diss_^e^ (kcal/mol)	Dissociation pattern^f^
VL/VH-HTT-HTT-VL/VH	20.3	6.8	− 50.1	4.5	VL/VH-HTT + VL/VH-HTT
VL/VH -HTT	11.0 g	2.5^g^	− 18.0^g^	3.9^g^	VL/VH + HTT
VH-HTT-HTT-VH	12.7	3.1	− 21.3	0.2	VH-HTT + HTT-VH
VH-HTT	7.0^g^	0.89^g^	− 6.5^g^	2.5^g^	VH + HTT
HTT-HTT	2.7	0.79	− 5.6	1.1	HTT + HTT

a VL/VH: the variable domains of the light and heavy chains of C4 scFv; HTT: HTT(1-17).

b Total solvent-accessible surface area of the assembly.

c Total solvent-accessible surface area of the units of assembly buried upon formation of the assembly.

d The solvation free energy gain upon formation of the assembly, in kilocalories per mole.

e The free energy of assembly dissociation, in kilocalories per mole.

f Dissociation pattern for the calculated free energy of dissociation.

g Average value for both assemblies in the asymmetric unit.

## Footnotes

† http://www.ebi.ac.uk/pdbe/pisa/.

‡ www.pymol.org.
