# Supplementary material for: Structure of a Single-Chain Fv Bound to the 17 N-Terminal Residues of Huntingtin Provides Insights into Pathogenic Amyloid Formation and Suppression
Source: J Mol Biol. 2015 Jun 19;427(12):2166–78. doi: 10.1016/j.jmb.2015.03.021 (PMC4451460; doi:10.1016/j.jmb.2015.03.021)
Supplement: Supplementary file 1 — Supplementary material [file mmc1.pdf]

Structure of a Single-chain Fv Bound to the 17 N-Terminal Residues of Huntingtin Provides Insights into Pathogenic Amyloid Formation and Suppression

Erwin De Genst[1], Dimitri Y. Chirgadze[2], Fabrice A. C. Klein[3], David C. Butler[4, 5], Dijana Matak-Vinković[1], Yvon Trottier[3], James S. Huston[5], Anne Messer[4, 5], Christopher M. Dobson[1]

[1]Department of Chemistry, University of Cambridge, Lensfield Road, Cambridge CB2 1EW, UK

[2]Department of Biochemistry, University of Cambridge, Tennis Court Road, Cambridge CB2 1GA, UK

[3]Translational Medicine and Neurogenetics Programme, Institute of Genetics and Molecular and Cellular Biology, Illkirch Cédex, France

[4] Neural Stem Cell Institute, Regenerative Research Foundation, Rensselaer, NY 12144, USA; Department of Biomedical Sciences, University at Albany, Albany, NY 12208, USA.

[5]James S. Huston, The Antibody Society, Newton, MA 02462, USA

Correspondence to Erwin De Genst: ejjd2@cam.ac.uk

Supplementary material

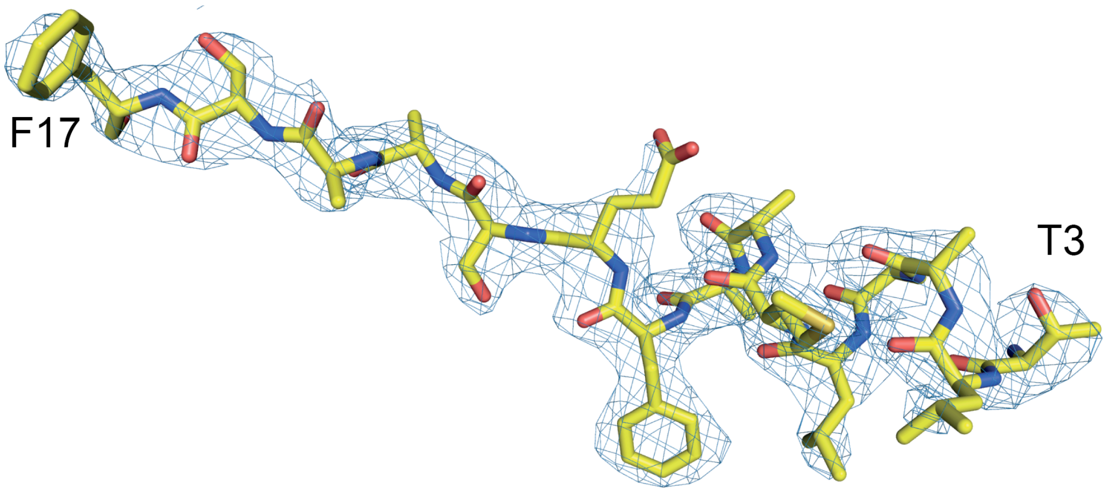

Figure S1. Electron density map for the HTT(1-17) peptide atoms in the C4 scFv:HTT(1-17) crystal structure. One of the HTT(1-17) peptides in the asymmetric unit, shown in stick representation. The atoms are color-coded: C, yellow; O, red; N, blue. The 2Fo-Fc map is contoured at 1.0 σ and represented as a blue mesh. The second peptide in the asymmetric unit has electron density of similar quality and definition.

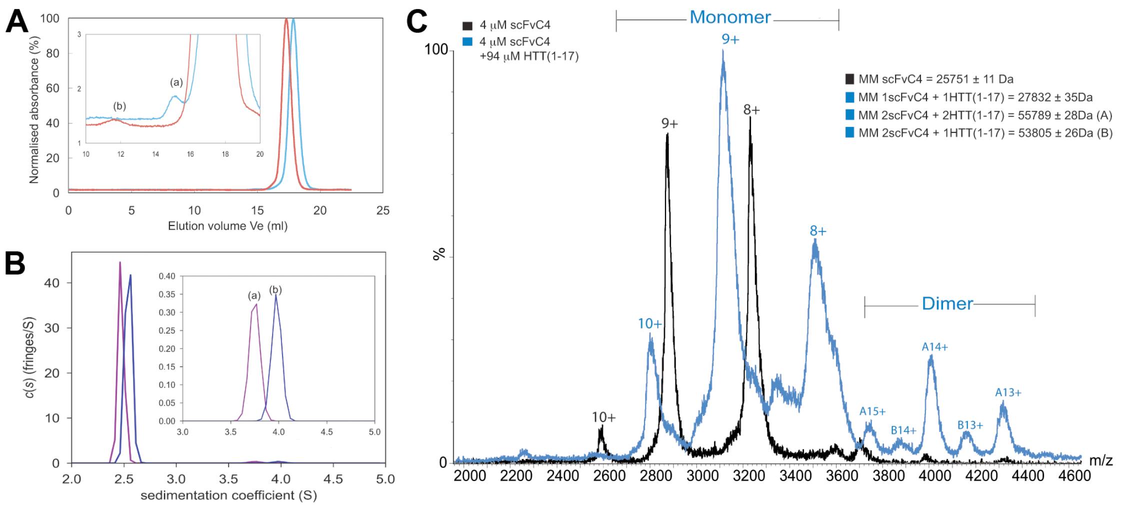

Figure S2. The C4 scFv:HTT(1-17) complex is predominantly monomeric in solution. A) Samples of C4 scFv alone and as an equimolar mixture with the HTT(1-17) peptide were subjected to analytical gel-filtration. The C4 scFv elutes as a globular protein of ~30 kDa The complex of C4 scFv with the peptide, resulted in a small decrease in retention volume consistent with a 1:1 complex of C4 scFv and HTT(1-17). The inset shows an expanded view of the chromatogram, showing the existence of an elution peak corresponding to a domain-swapped C4 scFv dimer [1], either free (peak (a) in the zoomed inset) or in complex (peak (b) in the zoomed inset) with the HTT(1-17) peptide. **B)** Analytical centrifugation experiments allowed higher resolution data and accurate estimates of the molecular masses for the observed species. The sedimentation analysis shows that samples of only C4 scFv contain predominantly a single species of molecular mass of ~25.8 kDa with a very small fraction of a protein species with a higher sedimentation coefficient (*s*), corresponding to a globular protein with a molecular mass around ~48.3 kDa (peak (a) in the zoomed inset of Figure S2B). These values are very close to the expected masses of the monomeric C4 scFv and its domain swapped diabody [1], with theoretical masses

of 25.6 kDa and 51.3 kDa. The samples of equimolar amounts of C4 scFv and the HTT(1-17) peptide contain protein species that sediment with slightly higher *s*-values than those observed for C4 scFv alone. The observed *s*-values correspond to molecular masses of ~27.8 kDa and ~54.5 kDa (the higher molecular weight species corresponds to peak (b) in the zoomed inset of Figure S2B), again comparing very well to the theoretical masses of 27.6 kDa and 55.2 kDa, for a 1:1 and a 2:2 complex of C4 scFv and HTT(1-17) **C)** Nondenaturing nanoelectrospray ionisation mass-spectrometry experiments, which enable the observation of non-covalent complexes that are present in solution. These experiments showed the presence of the same molecular species as those observed in the analytical centrifugation and gel filtration experiments. In the samples containing only C4 scFv we observed a dominant mass of 25,726 ± 21 Da and a dimeric peak of 51,603 ± 21 Da. The detection of the dimeric peak is, however, concentration dependent; it is visible at high concentrations of the C4 scFv but at a concentration of 4 μM this dimer peak can no longer be observed in the spectrum. Upon addition of increasing amounts of HTT(1-17) peptide to a sample of 4 μM C4 scFv, the monomeric form of C4 scFv becomes gradually saturated with peptide, and a dimeric peak also emerges corresponding to a complex of two C4 scFv proteins with two HTT(1-17) peptides. Although this dimer could correspond to the C4 scFv:HTT(1-17) complex found in the crystal structure, it is more likely that it represents a complex between a domain-swapped C4 scFv binding to two HTT(1-17) peptides, especially as we could only detect species that are consistent with such a complex, such as species with two scFv molecules bound to a single HTT(1-17) molecule.

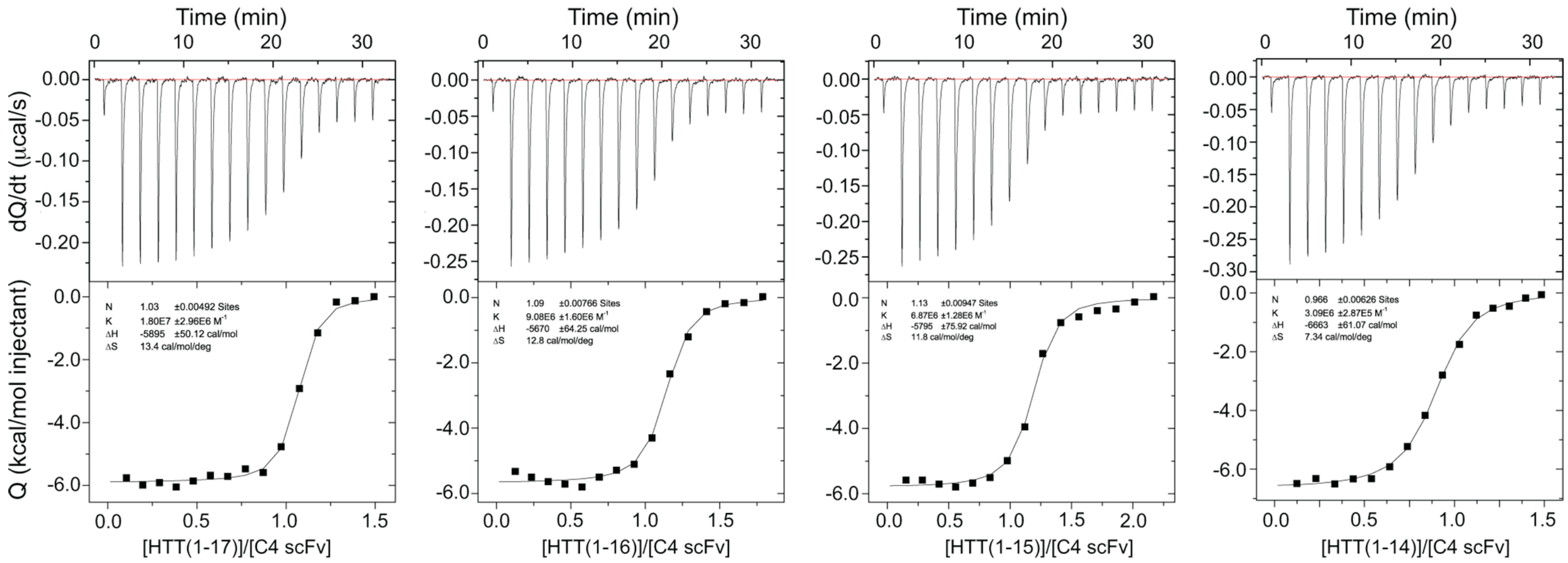

Figure S3. Isothermal titration calorimetry (ITC) measurements of HTT peptides binding to C4 scFv. ITC measurements for C-terminal truncated variants of the HTT(1-17) peptide. The top panels show the time course of the experiment, involving sequential injections of samples of the peptides into the ITC cell containing C4 scFv, resulting in bursts of heat release due to binding (μcal/s) and re-equilibration. The lower panels are plots of the integrated heat of each injection versus the ratio of titrant/analyte, and a least-squares fit to a simple Langmuir binding isotherm. The fitting procedure generates estimates for the number of binding sites "N" as well as the association constant for binding (Ka, M^{-1}) and the enthalpy change upon binding (ΔH, cal

mole^{-1}) and the entropy change upon complex formation (ΔS, cal mole^{-1} K^{-1}). These estimated values are plotted for each peptide on the graph.

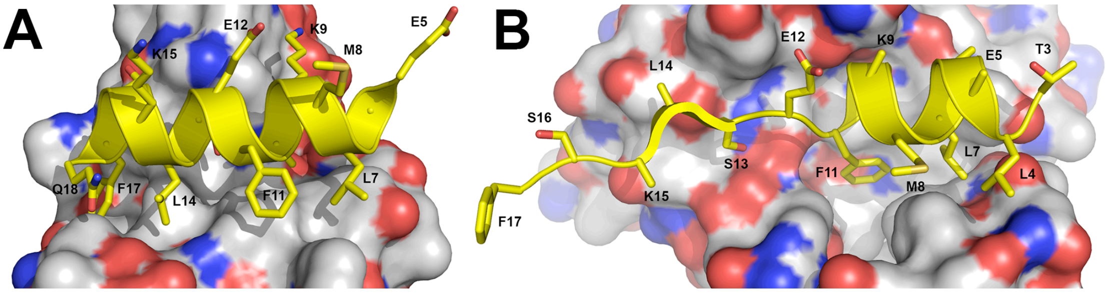

Figure S4. Comparison of the crystal structures of C4 scFv:HTT(1-17) and VL12.3:HTT(1-18). A) Crystal structure of VL12.3 in complex with HTT(1-17) Gln (PDB id: 3LRH) and **B)** the structure of C4 scFv in complex with HTT(1-17). The residues corresponding to the antibody fragments (e.g. VL12.3 or scFvC4) are represented as a continuous molecular surface, with C, N and O atoms, respectively, colored grey, blue and red. The peptide is drawn as a ribbon, with the atoms C, N and O, respectively, colored yellow, blue and red.

Table S1. List of interface residues in the crystal structure of C4 scFv (chain A (VH), B (VL), C (VH), D (VL)) in complex with HTT(1-17) (chain E,F)

Chain A	ASA/BSA, Å^2	Chain E	ASA/BSA, Å^2	Chain A	ASA/BSA, Å^2	Chain F	ASA/BSA, Å^2
S31	71.8/46.6*	T3	147.7/20.0	S31	71.8/3.7	E12	128.9/3.9
Y32	65.5/9.2	K6	62.7/18.0	Y53	119.9/22.3	L14	170.2/23.3
S33	31.6/31.2*	L7	98.9/37.5	D99	31.3/2.3	K15	103.9/8.6
S35	1.9/1.6	K9	59.5/34.9*	R100	183.5/94.7	S16	99.3/27.2
W47	82.5/17.1	A10	83.0/83.0*	Y101	135.7/2.3	F17	256.0/56.3
V50	40.2/39.8	F11	163.5/94.1*				
I51	2.6/0.8	E12	131.5/55.5*				
S52	21.4/21.2*	S13	107.7/66.3*				
Y53	119.9/50.8*	L14	108.6/57.1*				
N57	102.8/50.5*						
K58	88.5/8.0						
Y59	145.9/83.6						
D99	31.3/16.6*						
R100	183.5/30.7						
F102	63.4/8.1						

Chain B	ASA/BSA, Å^2	Chain E	ASA/BSA, Å^2	Chain B	ASA/BSA, Å^2	Chain F	ASA/BSA, Å^2
Y159	60.9/10.1	T3	147.7/3.1	Y161	121.6/50.1	S16	99.3/2.5
F220	79.9/41.6	L4	126.9/55.2	S219	4.2/0.3	F17	256.0/118.2
A221	10.7/0.6	L7	98.9/60.1	F220	79.9/38.2		
N222	121.6/49.1	M8	132.1/8.3	P225	92.4/11.9		
S223	110.6/5.72	F11	163.5/30.1				
G224	18.8/9.49						
P225	92.4/27.6						

Chain C	ASA/BSA, Å^2	Chain F	ASA/BSA, Å^2	Chain C	ASA/BSA, Å^2	Chain E	ASA/BSA, Å^2
S31	71.5/53.3*	T3	146.3/17.4				
Y32	65.2/7.3	K6	62.8/18.3	Y53	121/6.8	L14	108.6/6.7
S33	31.6/31.2*	L7	109.9/33.2	D99	34.3/4.6	K15	106.0/9.2
S35	1.6/1.2	K9	56.9/34.7*	R100	187.1/95.9	S16	111.6/27.3
W47	80.5/16.7	A10	85.7/85.7*	Y101	136.4/0.49	F17	254.5/58.0
V50	40.9/40.7	F11	166.0/103.6*	F102	62.7/1.65		
I51	2.5/0.3	E12	128.9/45.9*				
S52	20.5/19.7*	S13	106.6/66.6*				
Y53	121.0/57.0*	L14	170.2/71.3*				
N57	101.2/49.0*						
K58	90.2/7.9						
Y59	141.8/84.2						
D99	34.3/19.0*						
R100	187.1/29.3						
F102	62.7/9.9						

Chain D	ASA/BSA, Å^2	Chain F	ASA/BSA, Å^2	Chain D	ASA/BSA, Å^2	Chain E	ASA/BSA, Å^2
Y159	61.3/9.1	T3	146.3/2.3	Y161	125.6/50.9	S16	111.6/2.2
F220	77.2/41.3	L4	62.2/18.8			F17	254.5/116.1
A221	8.5/1.1	L7	109.9/68.9	F220	77.2/35.9		
N222	126.4/36.1	M8	131.5/26.0	P225	92.2/11.4		
S223	109.2/4.8	F11	166.0/27.0				
G224	19.8/9.3						
P225	92.2/27.9						

Chain F	ASA/BSA, Å^2	Chain E	ASA/BSA, Å^2
L7	109.9/7.8	L7	98.9/1.3
M8	131.5/38.7*	M8	132.1/48.8*
F11	166.0/35.4	F11	163.5/39.3
E12	128.9/36.0	E12	131.5/55.8
S13	106.6/34.5*	S13	107.7/35.9*
L14	170.2/45.9	L14	108.6/25.1
K15	103.9/54.2*	K15	106.0/55.1*
S16	99.3/51.9*	S16	111.6/60.7*
F17	256.0/81.5	F17	254.5/80.4

ASA – accessible surface area, BSA – buried surface area
* residue involved in interface hydrogen bond

Supplemental Materials and Methods

Size exclusion experiments. Size exclusion chromatography was performed using a Superdex 200 10/30 column (GE Healthcare, Buckinghamshire, UK) and an Akta Basic (GE Healthcare, UK). 100 μl samples of free C4 scFv (at 140 μM) or equimolar mixtures of C4 scFv and the HTT(1-17) peptide in PBS were injected and eluted from the column in PBS buffer at a flow-rate of 0.5 ml/min. Chromatograms were recorded by measuring the absorbance at 280 nm over one column volume starting from the injection of the sample.

Analytical centrifugation. Samples of HTT(1-17), C4 scFv and the C4 scFv:HTT(1-17) complex were prepared at equimolar concentrations in PBS buffer and dialysed extensively against PBS in the same sample container. Sedimentation velocity experiments were conducted with an Optima XL-I (Beckman-Coulter, High Wycomb, UK) centrifuge using an An60 Ti four-hole rotor. Standard double-sector Epon centrepieces equipped with sapphire windows contained 400 μL of C4 scFv, HTT(1-17) or an equimolar mixture of C4 scFv and HTT(1-17) at 1 mg/ml. Interference and absorbance (280 nm) data were acquired at 20°C and a rotor speed of 45,000 rpm, with systematic noise subtracted but without averaging and with radial increments of 0.003 cm. The density and viscosity of the buffer and the partial specific volume of the protein (based on the amino acid sequence) were calculated using Sednterp [2]. Multi-component sedimentation coefficient distributions were obtained using the program Sedfit v.14.1 (http://www.analyticalultracentrifugation.com/default.htm)

Mass spectrometry measurements. Nondenaturing nanoelectrospray ionisation mass spectra were recorded on a Synapt HDMS instrument (Waters, Manchester, UK), and calibrated using caesium iodide (100 mg ml^{-1}) using methodology described previously [3]. Typical values of MS parameters were: capillary voltage 1.8 kV, cone voltage 60 - 95 V, cone gas 40 L h^{-1}, extractor 1.0 – 1.3 V, ion transfer stage pressure 3.61 - 3.44 mbar, trap collision energy 20 V, transfer collision energy 15 V, trap and transfer pressure 5.29 – 5.33 × 10^{-2} mbar, IMS pressure 5.01 – 5.02 × 10^{-1} mbar, TOF analyser pressure 1.17 – 1.18 × 10^{-6} mbar. The program Micromass MassLynx 4.1 (Waters, Manchester UK) was used for data acquisition and processing.

Isothermal Calorimetry measurements. 2 mg of the peptides HTT(1-17), HTT(1-16), HTT(1-15) and HTT(1-14) (Insight Biotechnology Limited, Wembley, UK), were initially dissolved in 100 mM NaOH and then immediately neutralized with 100 mM HCl. The concentrations of these stock solutions were determined using standard ion-exchange-ninhydrin amino-acid analysis. The concentration of the C4 scFv was determined by UV absorption spectrometry at 280 nm using a molar extinction coefficient of 44,600 $M^{-1}cm^{-1}$, which was calculated based on the sequence of C4 scFv. Calorimetric data was obtained for the binding of C4 scFv to the peptides HTT(1-17), HTT(1-16), HTT(1-15) and HTT(1-14) in PBS and at 298 K using an Auto iTC200 calorimeter (MicroCal, Northampton, MA, USA). 40 µl solutions of the peptides, at concentrations 146 µM, 175 µM, 212 µM and 178 µM, for respectively HTT(1-17), HTT(1-16), HTT(1-15) and HTT(1-14), were titrated in

2.5 µl aliquots into the calorimetric cell containing a standard volume of 203 µl of a 20 µM monomeric C4 scFv solution in PBS. Injections of the solutions of the peptides into the sample cell were performed at 125 s intervals. The thermodynamic analysis was performed with Microcal analysis software (Origin 7.0) using a 1:1 bimolecular binding model.

References

[1] Arndt KM, Müller KM, Plückthun A. Factors influencing the dimer to monomer transition of an antibody single-chain Fv fragment. *Biochemistry* **37**, 1998, 12918–12926.

[2] Laue, T.M., Shah, B.D., Ridgeway, T.M., and Pelletier, S.L. 1992. Computer-aided interpretation of analytical sedimentation data for proteins. In *Analytical ultracentrifugation in biochemistry and polymer science* (eds. S.E. Harding et al.), pp. 90–125. The Royal Society of Chemistry, Cambridge, UK.

[3] Hernández H, Robinson CV. Determining the stoichiometry and interactions of macromolecular assemblies from mass spectrometry. *Nat Protoc* **2**, 2007, 715–726.
